# Irinotecan treatment and senescence failure promote the emergence of more transformed and invasive cells that depend on anti-apoptotic Mcl-1

**DOI:** 10.18632/oncotarget.2774

**Published:** 2014-11-06

**Authors:** Barbara Jonchère, Alexandra Vétillard, Bertrand Toutain, David Lam, Anne Charlotte Bernard, Cécile Henry, Sophie De Carné Trécesson, Erick Gamelin, Philippe Juin, Catherine Guette, Olivier Coqueret

**Affiliations:** ^1^ Paul Papin ICO Cancer Center, INSERM U892, CNRS 6299, Angers University, Angers, France; ^2^ INSERM U892, CNRS 6299, Nantes University, Nantes, France; ^3^ Gauducheau ICO Cancer Center, INSERM U892, CNRS 6299, Nantes, France

**Keywords:** Chemotherapy, senescence, irinotecan, drug resistance, colorectal cancer

## Abstract

Induction of senescence by chemotherapy was initially characterized as a suppressive response that prevents tumor cell proliferation. However, in response to treatment, it is not really known how cells can survive senescence and how irreversible this pathway is. In this study, we analyzed cell escape in response to irinotecan, a first line treatment used in colorectal cancer that induced senescence. We detected subpopulations of cells that adapted to chemotherapy and resumed proliferation. Survival led to the emergence of more transformed cells that induced tumor formation in mice and grew in low adhesion conditions. A significant amount of viable polyploid cells was also generated following irinotecan failure. Markers such as lgr5, CD44, CD133 and ALDH were downregulated in persistent clones, indicating that survival was not associated with an increase in cancer initiating cells. Importantly, malignant cells which resisted senescence relied on survival pathways induced by Mcl-1 signaling and to a lesser extent by Bcl-xL. Depletion of Mcl-1 increased irinotecan efficiency, induced the death of polyploid cells, prevented cell emergence and inhibited growth in low-adhesion conditions. We therefore propose that Mcl-1 targeting should be considered in the future to reduce senescence escape and to improve the treatment of irinotecan-refractory colorectal cancers.

## INTRODUCTION

Conventional genotoxic treatments induce cell death through apoptosis or senescence, a long term cell cycle exit where cancer cells remain viable but proliferation is definitely arrested. This response relies on the p53-p21waf1 and p16INK4-Rb pathways that prevent cell cycle progression and lock E2F-responsive genes in an inactive stage. Initially characterized in primary cells, animal models and human biopsies have demonstrated that senescence limits tumor progression and is necessary for a chemotherapy response [1-5]. Cell cycle arrest and senescence should be distinguished [6], first because senescent cells loose definitely their replicative potential, which is not always the case during the inhibition of cell cycle progression. In addition, senescence is also viewed as a consequence of cell cycle arrest that occurs when cells are overwhelmed at the same time by growth-promoting pathways and inaproppriate mTOR signaling [7-10].

In response to treatment, it is however unclear if senescence is always irreversible and why or how some cells preserve their proliferative potential in response to this suppressive mechanism. Theoretically, clones can exit senescence by the acquisition of secondary mutations such as p53 inactivation, but this is difficult to reconcile with the absence of proliferation of treated cells. Interestingly, several studies have reported non mutational mechanisms of drug resistance. For instance, a small subpopulation of «cancer-initiating cells» (CICs) endowed with an intrinsic drug resistance program represents a major source of therapeutic failure in breast cancer [11-13]. These initiating cells exist in a dynamic equilibrium with a differentiated counterpart, they auto-regenerate and thus reconstitute a complete population in response to stressful conditions [14]. The epithelial-mesenchymal transition (EMT) plays an important role in this phenotypic adaptation through the conversion of epithelial to mesenchymal cells endowed with CIC properties [11, 15-17]. In lung cancer, subpopulations of persistent cells can also resume proliferation when the majority of the population dies in response to EGFR tyrosine kinase inhibitors. This has been associated with an altered chromatin state mediated by the RBP2/KDM5A/Jarid1A histone demethylase [18]. Such adaptive and reversible mechanisms have also been described in bacteria where the rate of switching between sensitive and resistant bacteria explains how a total population can ultimately be reconstituted when antibiotic treatment is released [19].

We have recently shown that senescence escape and persistence also occur in colorectal cancer in response to the oncogenic stress mediated by Rasv12 [20]. Persistence was associated with p21waf1 down-regulation, increased genomic instability and with a dependency on Bcl-xL/Mcl-1 prosurvival signaling. In the current study, we pursued these experiments on senescence escape in response to chemotherapy. In unresectable metastatic colorectal cancer, irinotecan is used as a first or second line treatment in the FOLFIRI (leucovorin/5-FU/irinotecan) regimen. Unfortunately, tumor progression occurs generally rapidly [21], indicating that some cells can adapt to this topoisomerase I inhibitor. Several resistance mechanisms have been described, such as drug transport and metabolism, enhanced DNA repair and compensatory feedback pathways [22]. In this study, we described that a small population of cells can escape senescence and adapt to irinotecan treatment. Emerging within an heterogeneous population, persistent cells appeared to be more transformed and invasive than the parental population because they acquired the ability to grow in low-adherence conditions and to proliferate within a matrigel matrix. Interestingly, these cells show dependence on Mcl-1 signaling, probably as a consequence of pro-apoptotic pathways activated during senescence resistance.

Therefore, although chemotherapy killed off the vast majority of the initial population, some cells resist to this treatment and afterwards emerge as more aggressive cells. Besides the acquisition of secondary mutations, we propose that this adaptation mechanism plays an important role in the response failure of colorectal cancer to topoisomerase I inhibitors.

## RESULTS

### Various cell outcomes in response to topoisomerase I inhibition

**Figure 1:**
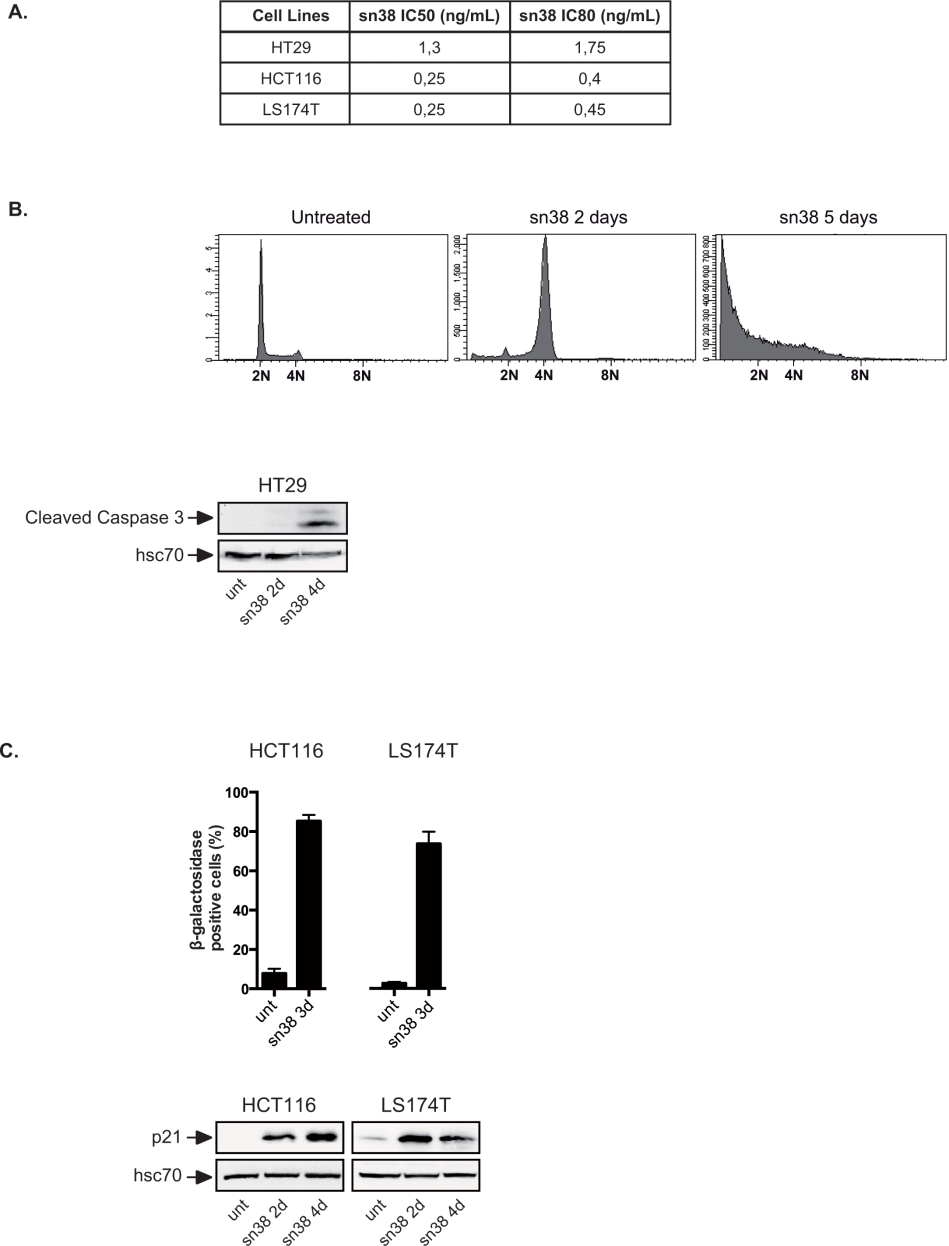
Topoisomerase I inhibition leads to apoptosis or senescence A. Clonogenic assays of colorectal HT29, HCT116 and LS174T cells. Cells have been treated with different doses of sn38 for 7 to 10 days and colony formation was then counted using crystal violet coloration. For each cell line, the growth of non treated cells was set up at 100% and IC50 and IC80 doses were determined (n=3). B. HT29 cells have been stimulated or not with sn38 (5ng/ml) for the indicated times and flow cytometry experiments were performed to quantify the number of subG1 cells using DAPI staining. In parallel, total cell extracts were recovered and caspase 3 activation was evaluated by western blot analysis (n=2). C. HCT116 and LS174T have been stimulated or not by sn38 (5ng/ml) as indicated. The percentage of senescent cells was evaluated as the number of cells expressing SA-ßgal activity (n=3 +/− sd) and evidenced by the expression of p21waf1 by western blot.

To determine whether colorectal cells can tolerate chemotherapy, three different cell lines were treated with sn38, the active metabolite of irinotecan. LS174T and HCT116 cells express a functional p53-p21 signaling pathway and HT29 cells were used as an example of cells expressing an inactive form of the p53 tumor suppressor. Using first clonogenic assays, we observed that LS174T and HCT116 have equivalent sensitivity to the topoisomerase inhibitor whereas HT29 cells were slightly more resistant (Figure 1A). sn38 was then used at 5 ng/ml because this concentration prevented cell proliferation of the three cell lines in clonogenic conditions. Note that pharmacokinetic studies have shown that this also corresponds to the plasma concentration of sn38 [23]. Using HT29 as a first experimental model, we observed that cells arrested after two days of treatment with a 4N DNA content and then entered apoptosis as evidenced by the presence of subG1 cells and caspase 3 activation (Figure 1B). This was expected since apoptosis is the main response to DNA damaging drugs when p53 is inactivated [24-27]. These experiments were then repeated in HCT116 and LS174T cells that express a functional p53-p21 pathway. As expected, the p21waf1 cell cycle inhibitor was expressed in both cell lines in response to sn38 and senescence was induced, as evidenced by standard beta-galactosidase assays (Figure 1C). By contrast, caspase 3 or the presence of subG1 cells were not detected in these conditions (data not shown). We then focused on these two cell lines.

**Figure 2:**
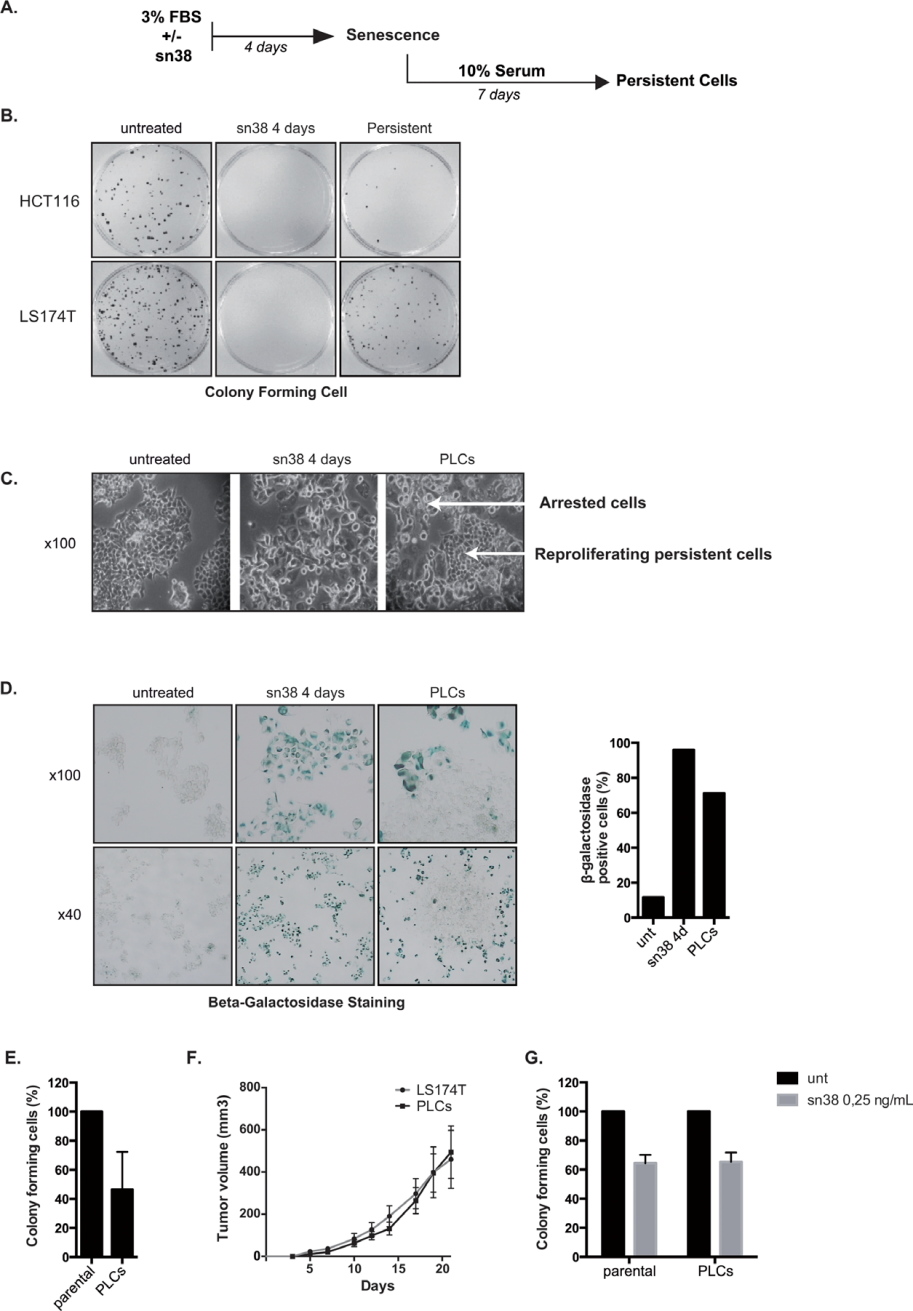
Cell emergence following sn38 treatment A. Experimental procedure to generate persistent cells. Cells have been treated or not with sn38 (5ng/ml) in 3% FBS for 4 days and then further stimulated with 10% FBS for 7 days to reinduce cell growth in the absence of drug. To measure cell proliferation, clonogenic assays were then performed using either untreated cells, cells treated for 4 days or persistent cells. B. Representative images of the proliferation capacity of the different cells. Untreated cells, cells treated for 4 days with sn38 and persistent cells were recovered and analyzed using clonogenic assays (representative image of three experiments). C. Representative images of PLCs heterogeneity. Note the emergence of islets of proliferating cells in PLCs (KI67 staining is presented Figure 7, representative image of four experiments). D. Representative images of cells expressing SA-ßgal activity in untreated cells, after 4 days of treatment or in PLCs. Quantification of one representative experiment is shown on the right. E. The proliferative capacity of parental LS174T cells and PLCs was quantified by clonogenic test (n=3+/−sd). F. *In vivo* evaluation of tumor formation by parental LS174T cells or PLCs. Cells were injected subcutaneously in immunocompromised mice and the tumor volume was monitored during twenty days ( 6 mice were used per condition in each experiment). G. sn38 sensibility of parental LS174T cells and of PLCs was evaluated by clonogenic assays using IC50 concentration (n=3+/−sd).

Although senescence is considered as a definitive arrest of cell proliferation, how cells escape this suppressive mechanism in response to chemotherapy has not been explained in detail. To determine if/how some cells preserve their proliferative capacities in response to senescence-inducing treatments, LS174T and HCT116 cells were stimulated with 10% serum after 4 days of sn38 treatment (see Figure 2A). The ability to proliferate was then evaluated by clonogenic assays, in untreated cells, after 4 days of treatment or in surviving cells (day 11) and representative images are presented Figure 2B. After 11 days, results showed that some LS174T clones resumed proliferation whereas most HCT116 cells remained essentially arrested at that time (Figure 2B). LS174T clones that emerged from senescence will be named persistent LS174T cells (PLCs). Visual microscopic analysis showed that PLCs emerged as a mixed subpopulation of cells that were either arrested or proliferating (Figure 2C, see also below Figure 7 for KI67 staining). Beta-galactosidase staining indicated that around 70% of PLCs were still senescent, whereas 20-30% of the total population had restarted proliferation at that time (Figure 2D). Clonogenic tests showed that PLCs divided less efficiently than parental LS174T cells (Figure 2E). To extend this observation, PLCs were injected subcutaneously in immunocompromised mice and their ability to form tumor was compared to that of parental LS174T cells. Surprisingly and despite the fact that PLCs were composed of 70% senescent cells, tumor formation was equivalent between parental cells and PLCs (Figure 2F). We then questioned whether we had selected clones that possessed an intrinsic resistance to the treatment. Clonogenic tests were performed to compare the sensitivity of the persistent cells to the parental LS174T population. Results indicated that PLCs had the same sn38 IC50 sensitivity as compared to the parental population (Figure 2G). Note however that differences in proliferation rate (Figure 2E) and drug sensitivity (Figure 2G) are difficult to interpret due to the presence of different populations within the PLCs.

Altogether, these results indicate that colorectal cells respond to topoisomerase I inhibition with different outcomes, entering either apoptosis or senescence. Among these, a subpopulation of LS174T cells can survive and emerge within an heterogeneous subpopulation to resume proliferation and form tumors *in vivo*.

### sn38 treatment induced the appearance of cells that resist to anoikis

**Figure 3:**
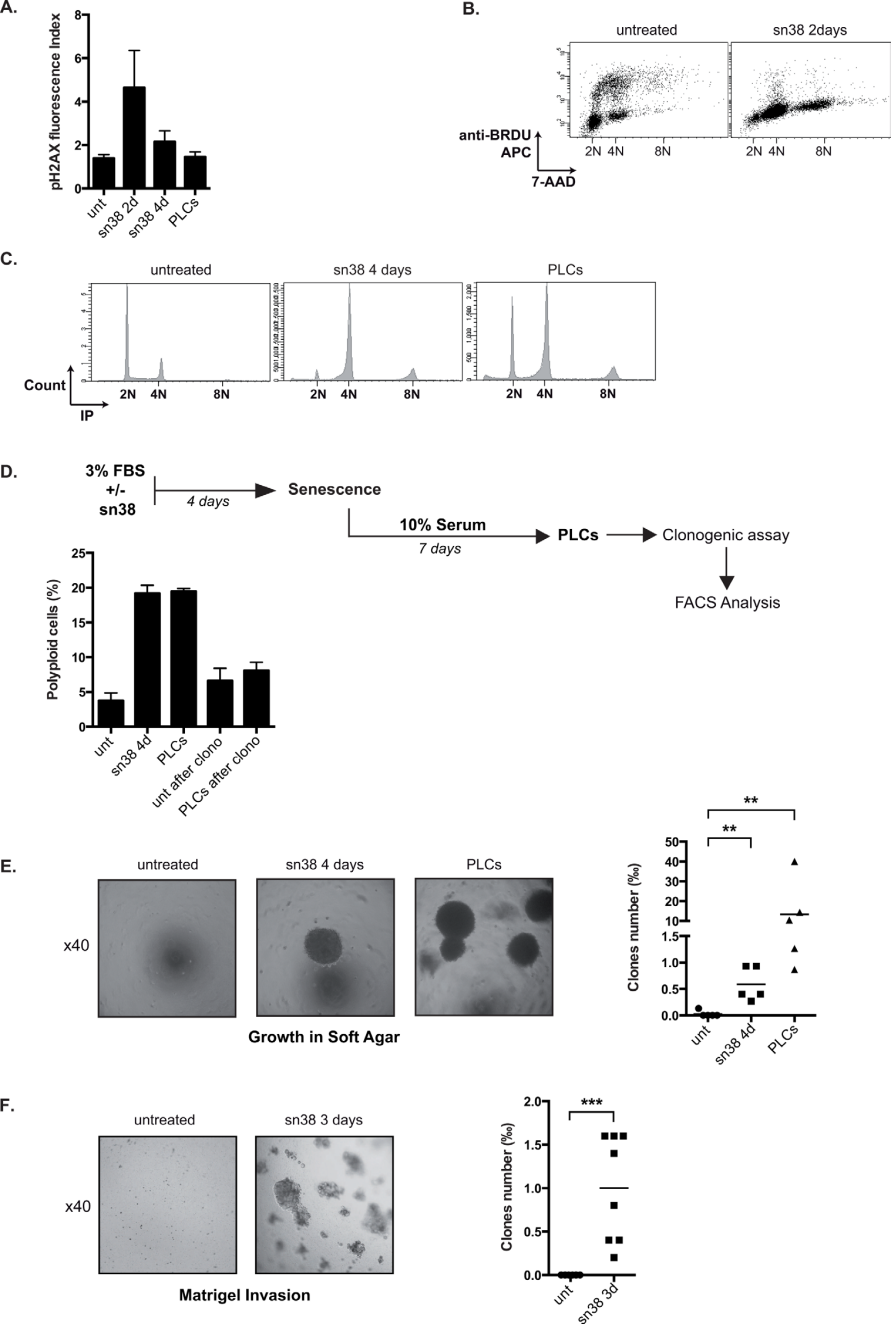
PLCs are more transformed as compared to parental cells A. DNA damage has been evaluated following treatment and in PLCs by FACS analysis using an antibody directed against the serine 139 phosphorylated form of H2Ax, the mean of fluorescence is presented (n=4 +/−sd). B. LS174T cells have been treated or not with sn38 (5ng/ml) for 48 hrs in the presence of BrdU. Cells were then analyzed for cell cycle distribution by flow cytometry using BrdU labeling and 7AAD staining (representative image of three experiments). C. Vindelov 83 coloration by flow cytometry has been performed to analyze cell cycle profiles and polyploidy in the different conditions (one image is shown, representative of three different experiments). D. Analysis of the proliferative capacity of polyploid cells. LS174T cells treated with sn38 (5ng/ml) for 4 days were further stimulated with 10% FBS for 7 days to reinduce cell growth and generate polyploid cells. Cells were then trypsinized, replated in 10% FBS and allowed to form colonies for 7-10 days. The presence of polyploid cells was then analyzed by flow cytometry, using either parental cells (unt. after clono.) or PLCs for this procedure (PLCs after clono. n=3+/−sd). E and F. Growth of the PLCs was evaluated in soft agar (E) or in matrigel (F), note that 5000 cells were used for these experiments. Representative images are shown (x40) and the number of clones growing in low adhesion has been quantified after 10-20 days (n=5 for soft agar and 8 for matrigel).

We were intrigued by the fact that PLCs induced *in vivo* tumor formation to the same extent as parental cells despite the fact that they were essentially composed of senescent cells. We therefore determined if sn38 escape induced the emergence of cells that were more transformed and aggressive than parental cells. Using serine 139 phosphorylation of histone gamma-H2Ax as a marker of DNA double strand breaks, we observed by flow cytometry that sn38 induced DNA damage after two days as expected (Figure 3A). H2Ax phosphorylation returned to basal levels in PLCs, suggesting that DNA repair occurred efficiently. However, a significant amount of polyploid LS174T cells was detected after two days (Figure 3B), and these cells remained viable since they were detected after 4 days and in the PLCs (Figure 3C). To determine if these abnormal cells were dividing, clonogenic assays were performed using PLCs and DNA content was analyzed by FACS at the end of the assay (see Figure 3D). Cells with polyploid DNA were not detected at the end of the clonogenic tests, indicating that these cells are probably growth arrested within the heterogeneous PLC population (Figure 3D).

We then used growth in soft agar as an indicator of anoikis resistance and increased cell transformation. To this end, cells were grown in 3% FBS for four days in the presence or absence of sn38, cells were then trypsinized, transferred to soft agar and further grown for 10-20 days. We observed in this condition that the survival of parental LS174T cells was significantly reduced (see materials and methods). By contrast, a subpopulation of sn38-treated cells was able to grow in soft agar after 4 days of treatment (Figure 3E). PLCs were also able to survive in these low-adhesion conditions. Since the same number of cells were plated in soft agar as compared to parental cells, this result indicates that abnormal cells were generated during the early response to sn38 and that they were not pre-existent. These experiments were then repeated using a Matrigel matrix where parental LS174T cells were not able to survive. Again, a subpopulation of cells was able to proliferate and invade this matrigel matrix during the early response to sn38 (Figure 3F).

Therefore, we concluded from these results that following sn38 treatment, a small subpopulation of LS174T persistent cells emerged as more transformed cells that were able to grow in low-adhesion conditions.

### Salinomycin increased the number of PLC and anoikis resistance

**Figure 4:**
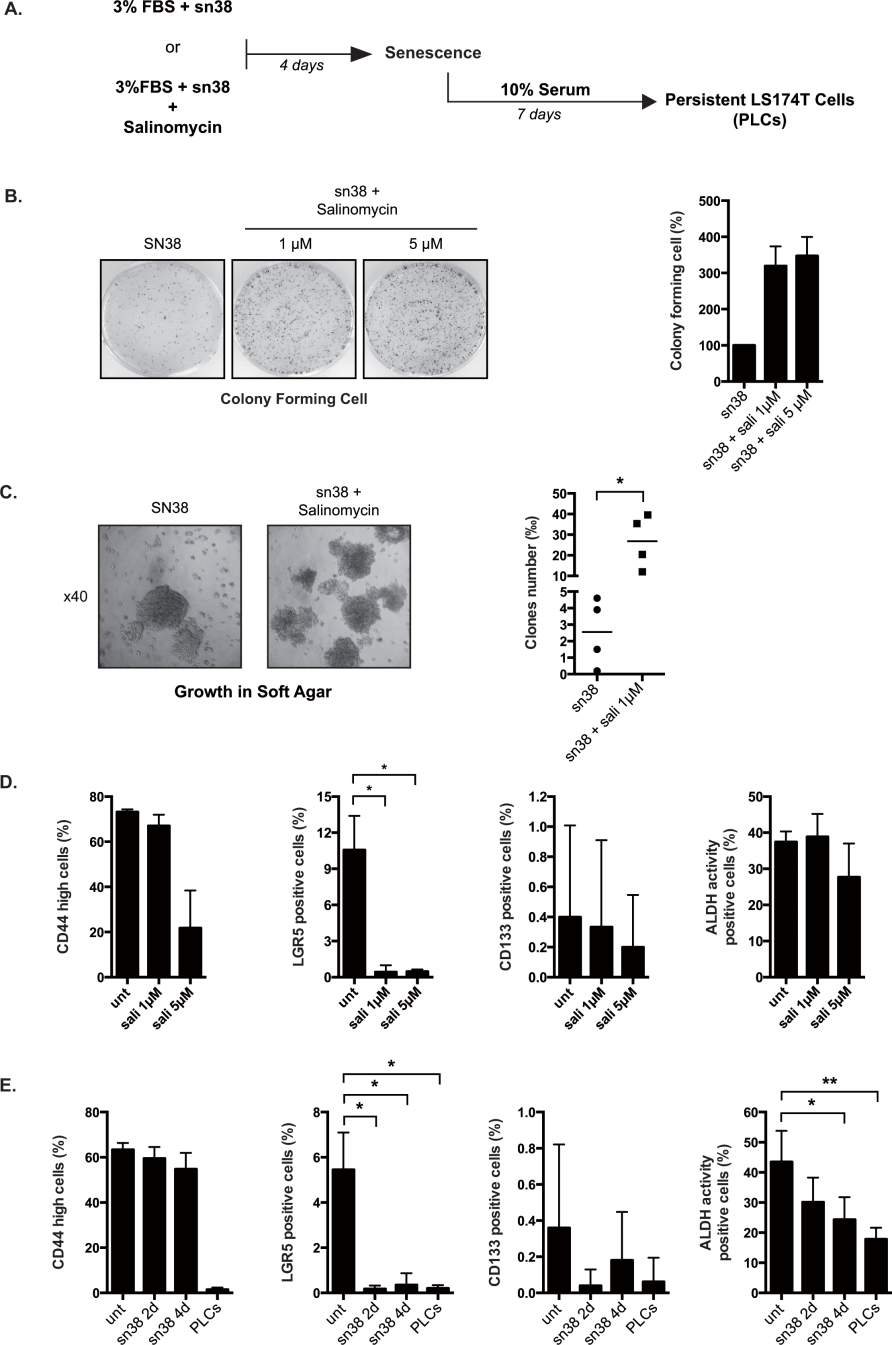
Salinomycin enhances emergence and growth in soft agar A. Cells have been treated or not with sn38 (5ng/ml) for 4 days in the presence or absence of salinomycin (1 and 5μM). Cells were then further stimulated with 10% FBS for 7 days to reinduce cell growth. B. Cells have been treated with sn38 in the presence or absence of salinomycin and the proliferative capacity of PLCs was evaluated by clonogenic tests. Representative images are shown on the left and the quantification of clonogenic results is presented on the right of the figure. Salinomycin was used at 1μM (n=3+/−sd) or 5 μM (n=2 +/− sd). C. LS174T cells have been treated or not with sn38 and salinomycin (1μM) for 3 days. Growth in soft agar was evaluated for 10-20 days. 5000 cells were used in each experiment. Representative images are shown (x40) and the number of clones growing in low adhesion was quantified (n=4). D. CD44^high^, lgr5, CD133 expressions and Aldh activity have been analysed by flow cytometry following salinomycin treatment of parental LS174T cells for 4 days in 10% FBS. Percentage of positive cells are presented (n=3 +/−sd). E. FACS analysis was performed in cells treated or not with sn38 or in PLCs as indicated (n=3 +/−sd).

Chemotherapy escape has been associated with the survival of a subpopulation of cancer initiating cells (CIC) that possess an intrinsic resistance to genotoxic treatment. Using a chemical screen, Gupta et al. have recently identified drugs such as salinomycin that specifically kill these initiating cells [28]. Combinatorial therapy targeting at the same time CICs and differentiated cancer cells are expected to be more efficient than chemotherapy alone [13, 29]. If CICs allowed survival and PLCs emergence, we reasoned that salinomycin should improve the efficiency of sn38. To verify this hypothesis, we added salinomycin together with sn38 at the beginning of the treatment and cell emergence was then evaluated (see Figure 4A). Surprisingly, results showed that salinomycin significantly enhanced the number of proliferating PLCs (Figure 4B) and the number of cells growing in soft agar (Figure 4C).

We then determined whether salinomycin effectively reduced the expression of the main proposed markers of colorectal cancers stem cells, LGR5, CD133, CD44 and ALDH [30, 31]. Using flow cytometry, a decrease of CD44^high^ and LGR5 expression was observed in response to salinomycin. No significant effect was noticed on CD133 expression and ALDH activity (Figure 4D). We then analyzed the expression of CD44, LGR5, CD133 and ALDH activity in PLCs. Results presented Figure 4E indicate that these markers were down-regulated in the persistent cells.

Altogether, these results indicate that senescence escape and persistence were not associated with an increase in cancer initiating cells. In addition, salinomycin, although known to target breast CICs, presented unexpected effects in colorectal cells when added with sn38, by increasing the proportion of cells that were able to resist and grow in soft agar.

### Mcl-1 and Bcl-xL prevented cell death during the early response to sn38

**Figure 5:**
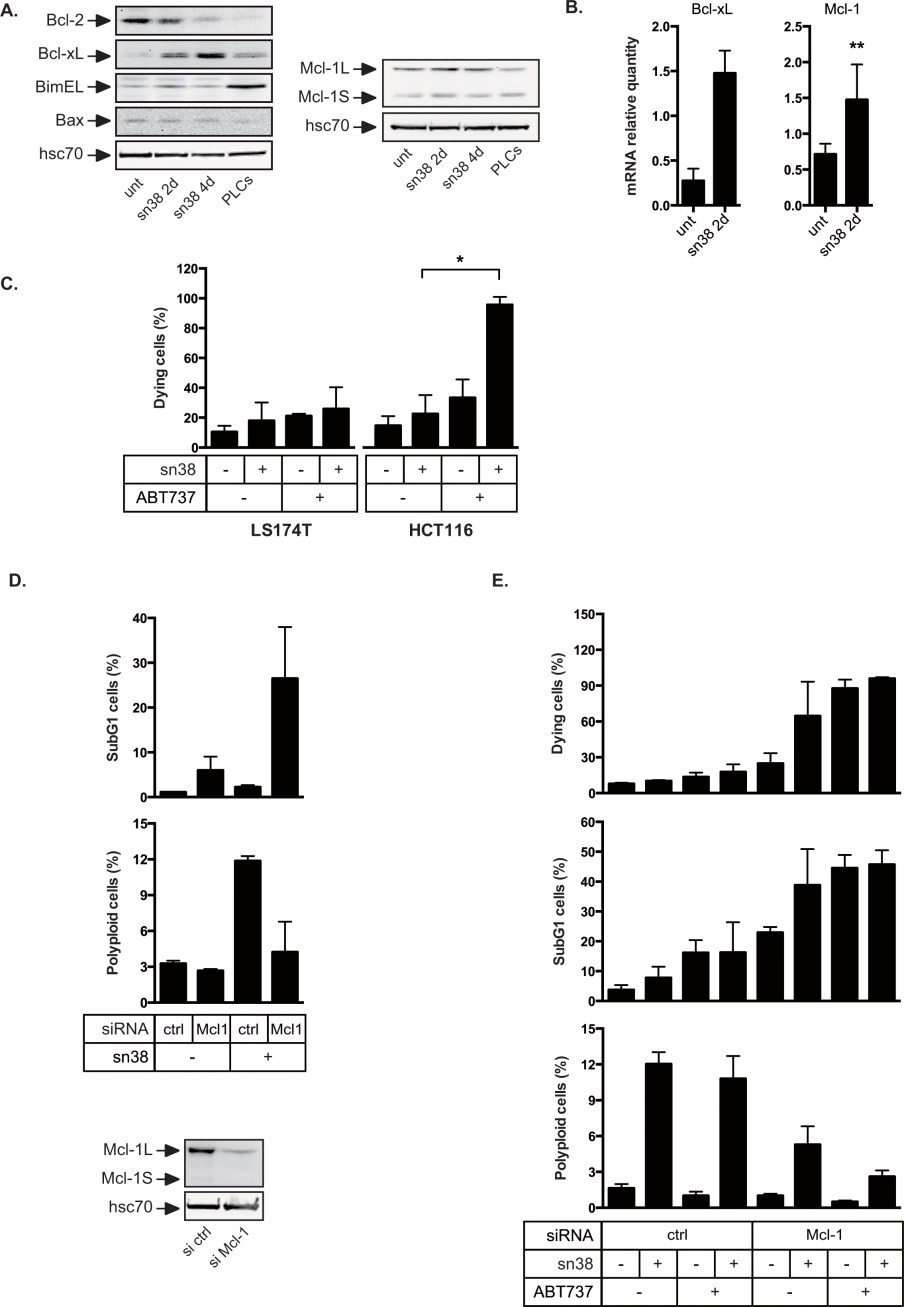
Overexpression of Bcl-xL and Mcl-1 in response to sn38 A. Bcl-xL, Bcl2, Mcl-1, Bim, Bax, and hsc70 expressions have been evaluated by western blot analysis using total cell extracts, following sn38 treatment or in PLCs (n=3). B. The mRNA expressions of Bcl-xL and Mcl-1 have been evaluated by quantitative RT-PCR (n=3 for Bcl-xL and n=5 for Mcl-1). C. LS174T or HCT116 cells have been treated or not by sn38 for 3 days followed by the addition of ABT737 as indicated for 1 day. Cell death was then analyzed by trypan blue exclusion. Note that the early death to sn38 is evaluated here as opposed to long term clonogenic assays shown in Figures 1 and 2 (n=3 +/− sd). D. Mcl-1 expression was down-regulated by RNA interference and 1 day after LS174T cells have been treated with sn38 for 2 days. Cell death was evaluated by flow cytometry analysis through the evaluation of subG1 cells (n=3 +/−sd). In parallel, the percentage of polyploid (>4n) cells has been analyzed (n=3 +/−sd). Mcl-1 down-regulation is shown on the bottom part of the figure. E. Mcl-1 has been down-regulated as described above for 1 day, cells were treated with sn38 for 3 days followed by the addition or not of ABT737 during 1 day. Cell death was evaluated by trypan blue exclusion or subG1 analysis by flow cytometry (n=3 +/−sd). In parallel, the percentage of polyploid cells has been analyzed (n=3 +/−sd ).

We have recently proposed that Bcl-xL and Mcl-1 allow resistance to oncogene-induced senescence [20]. To determine if PLCs emergence was associated with the induction of anti-apoptotic signals, we analyzed the expression of Bcl-2 pro-survival proteins following acute treatment or in PLCs. Western blot experiments showed that Bcl-2 levels decreased but that Bcl-xL was up-regulated in response to sn38 (Figure 5A). Though we found in a few experiments that Mcl-1 was slightly induced by sn38, its expression was generally not significantly up-regulated. Quantitative RT-PCR experiments showed an early induction at the mRNA levels (Figure 5B). To determine if Bcl-xL and Mcl-1 compensated pro-apoptotic signals, Bax, Puma, Noxa and Bim levels were evaluated since they represent the main targets of Bcl-xL and Mcl-1 [32, 33]. Although we were not able to detect any increase in Bax, Puma and Noxa expressions, a significant up-regulation of Bim was observed in PLCs (Figure 5A and data not shown). These results suggest that pro-apoptotic signals are active in PLCs and that the titration of Bim by survival proteins might be necessary during the process of cell emergence.

We then determined if cell survival relies on the expression of Bcl-xL and/or Mcl-1 during the acute response to sn38. To this end, we used ABT-737, a known inhibitor of the Bcl-2 and Bcl-xL proteins. Cells were treated with sn38 for 4 days, in the presence or absence of ABT-737 for the last 24 hours. As visualized by trypan blue exclusion experiments, LS174T cells were not sensitive to ABT-737 (Figure 5C). HCT116 cells, used as a control, demonstrated the efficiency of the Bcl-2 inhibitor since Bcl-xL was also up-regulated in this cell line (data not shown). This suggested that Bcl-xL signaling was not the main survival pathway activated in LS174T cells to allow early sn38 survival. Mcl-1 was then inactivated by RNA interference in this cell line and its down-regulation was verified by western blot analysis (Figure 5D, bottom). Interestingly, Mcl-1 inhibition increased cell death following sn38 treatment as evidenced by the presence of subG1 cells (Figure 5D, top). In addition, this also led to a reduced number of polyploid cells (Figure 5D, middle), suggesting that cells with abnormal DNA content relied on Mcl-1 signaling.

It has been recently shown that the up-regulation of Mcl-1 induced ABT737 resistance [34]. For this reason, we determined if we could enhance cell death by targeting both survival proteins simultaneously. To this end, Mcl-1 was inactivated by RNA interference, and cells were treated with sn38 for 3 days, in the presence or absence of ABT737 for an additional 24 hrs. As visualized by trypan blue exclusion and the analysis of subG1 cells, Mcl-1 inactivation induced cell death and this effect was enhanced by ABT737 to kill almost all cells (Figure 5E). Interestingly, the combination also enhanced the death of polyploid cells as compared to the inactivation of Mcl-1 only (Figure 5E, bottom).

Altogether, these results indicate that Mcl-1 and Bcl-xL are necessary to allow the survival of normal and polyploid cells during the early response to sn38.

### Dependency on Bcl-xL and Mcl-1 for anoikis resistance and PLC emergence

**Figure 6:**
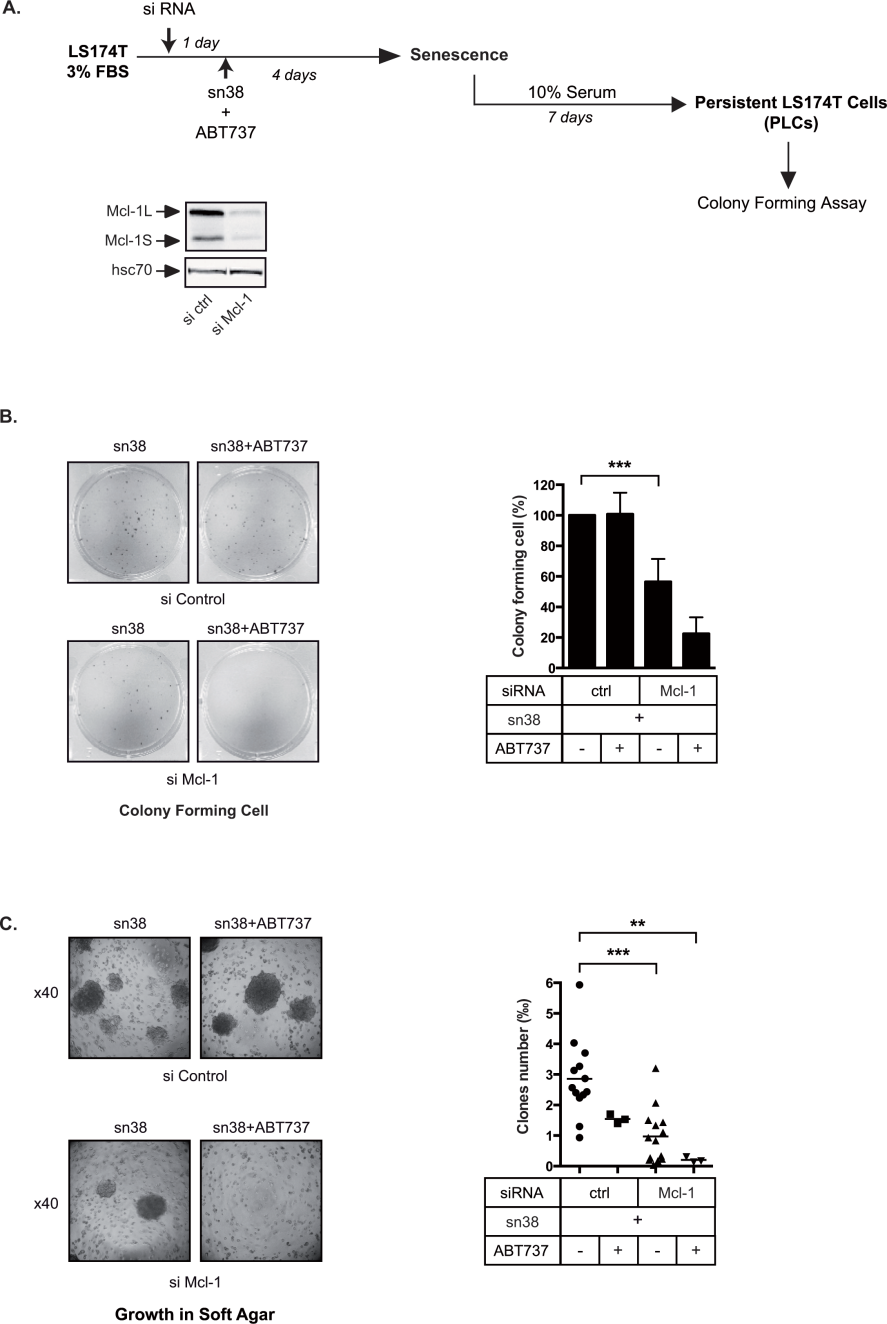
Persistent cells depend on Mcl-1 A. Experimental procedure to inactivate pro-survival proteins during the generation of persistent cells. Mcl-1 expression was down-regulated by RNA interference, 1 day after LS174T cells were treated with sn38 and, where indicated, with ABT737 for 4 days. Emergence and growth in low adhesion were then evaluated. B. Following Mcl-1 inactivation and ABT737 addition as indicated, the proliferative capacity of PLCs was evaluated by clonogenic tests (n=3). Representative images are shown on the left of the figure, and the quantification of clonogenic results are presented on the right (n=3 +/− sd). C. Following Mcl-1 inactivation and ABT737 addition as indicated, treated-cells were recovered after 4 days and grown in soft agar. Representative images are shown on the left of the figure (x40), and quantification results are presented on the right.

If Mcl-1 and Bcl-xL were necessary for the early survival in response to treatment, we reasoned that their inactivation should reduce growth in low adhesion and prevent PLCs emergence. To this end, Mcl-1 was inactivated by RNA interference and cells were then stimulated with sn38 in 3% FBS for 4 days. PLC proliferation was then evaluated as well as the ability to grow in soft agar. In the same experiments, we also tested the effect of the combined inactivation of Mcl-1 and Bcl-xL using ABT-737 (see Figure 6A). Clonogenic tests presented Figure 6B showed that Mcl-1 downregulation significantly reduced the number of proliferating PLCs. This effect was enhanced by ABT737, leading to a further reduction of the number of dividing cells. Mcl-1 inactivation also significantly prevented growth in soft agar as compared to a control siRNA (Figure 6C). Results showed that this effect was enhanced by ABT737, almost completely inhibiting growth in low adhesion (Figure 6C).

Altogether, these results indicate that Mcl-1 and Bcl-xL are necessary to allow PLC emergence and survival in low adhesion conditions.

### Senescent cells present within the PLC grew in low adhesion

**Figure 7:**
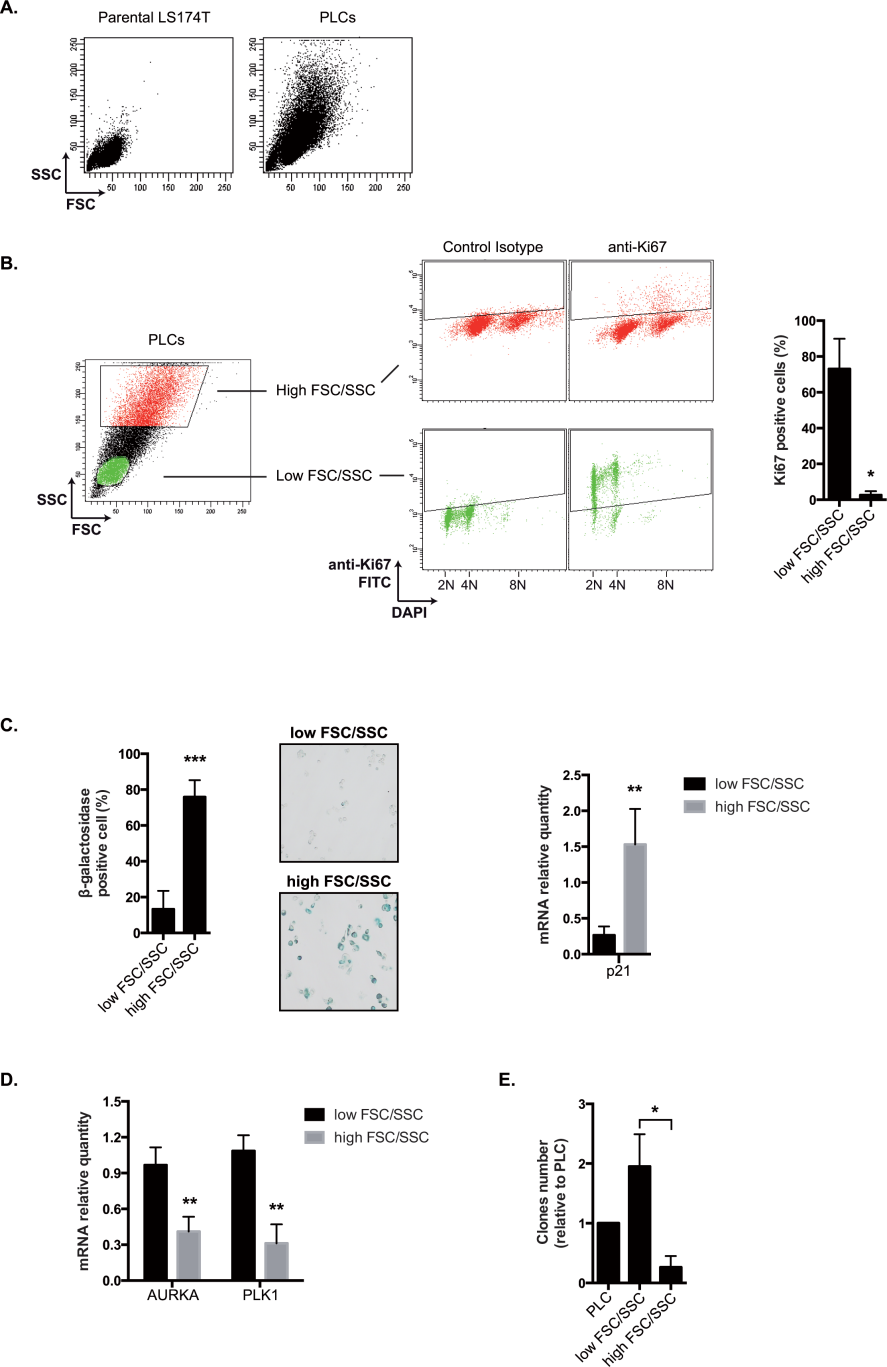
PLCs as an heterogeneous mixture of PLS and PLD cells A. SSC and FSC parameters have been evaluated by flow cytometry in parental LS174T cells and PLCs (one image representative of 6 experiments). B. Proliferation has been evaluated by flow cytometry using an antibody directed against the KI67 antigen. Following DNA DAPI staining, cells have been gated according to low or high FSC/SSC values and the corresponding KI67 expression has been evaluated. Percentages of positive cells are presented on the right part of the figure (n=4+/−sd). C. Cells have been cell sorted by flow cytometry according to low and high FSC/SSC parameters and the percentage of SA-ßgal positive cells has been evaluated in each subpopulation (n=8+/−sd). Representative images are shown on the middle part of the figure (x100). p21waf1 mRNA expression has been evaluated by quantitative RT-PCR in each subpopulation (n=5+/−sd). D. Cells have been cell sorted by flow cytometry according to low and high FSC/SSC parameters. Aurora-A and PLK-1 mRNA expressions in each subpopulation have been evaluated by quantitative RT-PCR (n=5+/−sd). E. Cells have been cell sorted by flow cytometry according to low and high FSC/SSC parameters. The proliferative capacity of the two subpopulations and of PLCs was quantified by clonogenic test (n=4+/−sd).

As described above Figure 2C/D, PLCs were heterogeneous and composed of around 70% senescent cells and 20-30% of proliferating cells. We therefore wanted to determine which subpopulation was able to survive in low adhesion and which cells expressed Mcl-1 and Bcl-xL. Senescent cells are known to increase in size and density as compared to normal counterparts [35]. Using flow cytometry analysis, we effectively detected increased cell size (forward scatter, FSC) and granularity (side scatter, SSC) in PLCs as compared to normal LS174T cells (Figure 7A). In addition, using FSC/SSC gating and KI-67 staining as a marker of cell proliferation, we noticed that a high FSC/SSC profile was associated with a significant decrease in KI-67 expression (Figure 7B). This suggested to us that this large subpopulation might contain the senescent cells. To test this hypothesis, PLC were sorted according to cell size and granularity and senescence was analyzed on each subpopulation using beta-galactosidase assays. Results presented Figure 7C indicated that 75,9%+/−8,8 cells with a high FSC/SSC profile stained positively for beta-galactosidase, whereas only 13,3%+/−10,2 cells with a low size and granularity were positive. Using RT-QPCR experiments, we also noticed that the former subpopulation overexpressed p21waf1 mRNA as compared to cells that had a lower FSC/SSC profile. On the opposite, these smaller non-senescent cells expressed higher level of Aurora-A and polo-like kinase 1 mRNAs, two mitotic kinases that we and other have shown to be downregulated in response to genotoxic treatments (Figure 7D) [36, 37]. Following cell sorting, clonogenic tests were then performed to evaluate the proliferative capacity of each subpopulation. Results presented Figure 7E indicated that cells with a low FSC/SSC profile divided more efficiently as compared to cells with a higher profile or to PLCs.

Altogether, these results suggest that we could separate PLCs according to their size and granularity to obtain enriched populations of senescent and dividing cells. The PLCs enriched in dividing cells (low FSC/SSC) will be named PLD whereas those enriched in senescent cells will be named PLS (high FSC/SSC).

**Figure 8:**
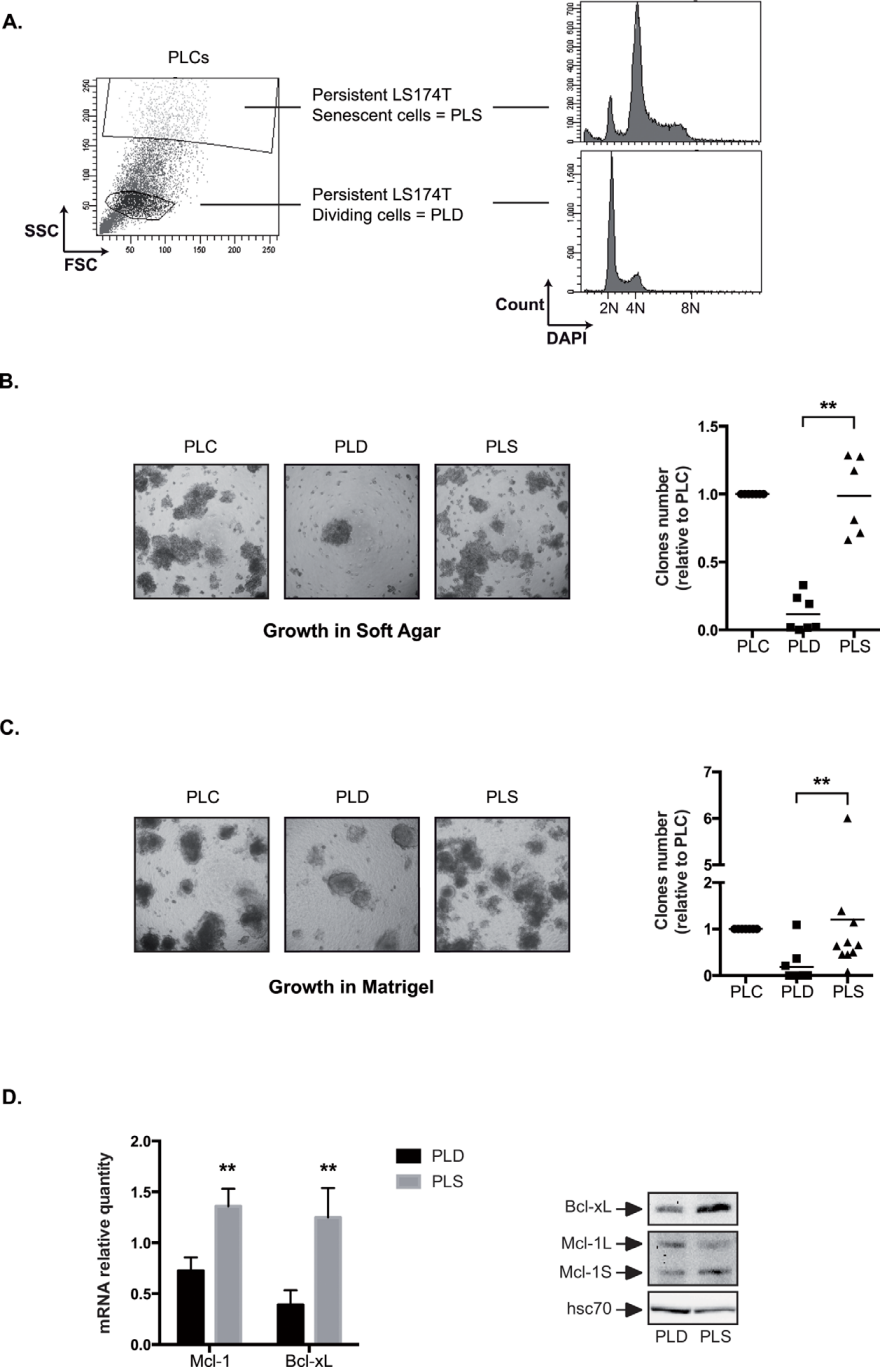
PLS cells grow in low adhesion conditions A. Following cells sorting and DAPI DNA staining after 3 days of culture, DNA content has been analyzed in PLD (enriched dividing cells = low FSC/SSC) and PLS (enriched senescent cells = high FSC/SSC) by flow cytometry (n=3, one representative image is shown). B and C. Growth of the PLD and PLS was evaluated in soft agar (B) or in matrigel (C). 10 000 cells were used in each experiment. Representative images are shown (x40) and the number of clones growing in low adhesion was quantified after 10-20 days (n=6 for soft agar and n=9 for matrigel). D. The mRNA and protein expressions of Mcl-1 and Bcl-xL have been evaluated by quantitative RT-PCR (n=5 +/− sd) and by western blot analysis (n=4) using total cell extracts of PLD or PLS cells as indicated.

Results presented Figure 3C showed that a significant amount of polyploid cells were present within the PLCs. Following cell sorting and DAPI staining, we observed that the cell cycle profile of PLD was normal whereas PLS presented an abnormal polyploid DNA content (Figure 8A). We then compared the growth in soft agar of the two subpopulations. To this end, PLCs were generated, PLD and PLS cells were cell sorted, and the enriched subpopulations were transferred to soft agar and further grown for 10-20 days. In these conditions, PLS cells were able to grow in soft agar whereas the number of growing PLD cells was significantly reduced (Figure 8B). The same observation was made when sorted cells where grown in matrigel. Whereas PLS proliferated within this matrix significantly, less PLD clones were able to grow in these low-adhesion conditions (Figure 8C). We then asked if PLD and PLS expressed to the same extent Mcl-1 and Bcl-xL. Using RT-QPCR and western blot experiments (Figure 8D), we observed that Bcl-xL was overexpressed in PLS as compared to PLD, both at the mRNA and protein levels. By contrast, Mcl-1L protein levels were slightly overexpressed in the PLD subpopulation.

Therefore, we concluded from these results that the ability to grow in soft agar and in Matrigel is due to the presence of senescent cells within the PLC emergent population.

## DISCUSSION

Colorectal tumors generally fail to respond to irinotecan within 8-9 months of treatment, indicating that some cancer cells adapt to this genotoxic drug [21]. We have previously described that Myc signaling and DNA repair pathways play an important role in chemotherapy resistance, notably when deregulated by the STAT3 oncogene [24, 36, 38, 39]. We now extend these results, showing that a small fraction of LS174T cells can tolerate this treatment, escape senescence and emerge as a dividing population. This adaptation was reversible since persistent cells regained drug sensitivity. In addition, cells with abnormal polyploid DNA content were present within the PLC population. Most importantly, PLCs were able to grow in low adhesion conditions and to proliferate within a matrigel matrix. Thus, although they regained sn38 sensitivity, these cells evolved to resist anoikis and become more aggressive. Since oncogenic progression is associated with an intrinsic drug resistance program [40, 41], a subpopulation of resistant cells already present before the treatment could be theorically responsible for senescence resistance and reproliferation. However, we believe that a selection was unlikely to occur, since the low proliferation rate during the initial induction of senescence is not expected to favor mutagenesis and since the final population switched back to the same sn38 sensitivity. In addition, growth in low adhesion was observed very early during the treatment, in the absence of cell division (Figure 3E/F). Since parental cells did not grow in soft agar in our experimental conditions (3% FBS), this further indicates that the selection of a preexisting clone was unlikely to occur. In mammary epithelial cells, it has been proposed that cell cycle arrest and p21waf1 overexpression lead to anoikis resistance [42, 43]. This suggests that growth in low adhesion might be a common feature of the early response to genotoxic treatments, as a consequence of growth arrest and senescence induction. Since, anoikis resistance relies on Erk up-regulation and Bim inactivation, it will be interesting to determine the role of the Erk pathway during the escape to sn38.

We also observed that PLCs emerged as an heterogeneous population, composed of dividing (PLD) and senescent cells (PLS). This leads to the hypothesis that cells entered cell cycle arrest but that a few clones exit this suppressive pathway by a phenotypic switch that reconstitutes a complete dividing and sensitive population. As stated above, this adaptive mechanism has already been described in bacteria where so-called persister bacteria resist antibiotic treatment and reconstitute a full population which is again sensitive to the same antibiotic [19]. The manner in which this phenotypic adaptation occurs remains to be determined but it does not rely on any genetic change. Interestingly, this mechanism has been described recently in lung cancer, showing that equivalent persistent cells exist in human cancer and can tolerate chemotherapy [18]. Other results have reported that breast cancer stem cells and their differentiated counterparts exist in a dynamic equilibrium and that the conversion between the two cell types is regulated by inflammatory cytokines [14]. Further experiments are therefore necessary to clarify the interactions between the PLS and PLD and the relations between the subpopulations. It has been previously reported that senescent cells can stimulate cancer cell growth and invasion through a secretory phenotype and the production of matrix metalloproteinases [35]. In line with these observations, our results indicate that PLS grew in low adhesion conditions whereas this was not the case of the dividing subpopulation. It is tempting to speculate that PLS, through the production of an «invasive» secretome, provide a surviving niche where PLD cells could divide. Future experiments are necessary to determine if a cooperation between these two subpopulations is necessary to sustain an invasive phenotype.

**Figure 9:**
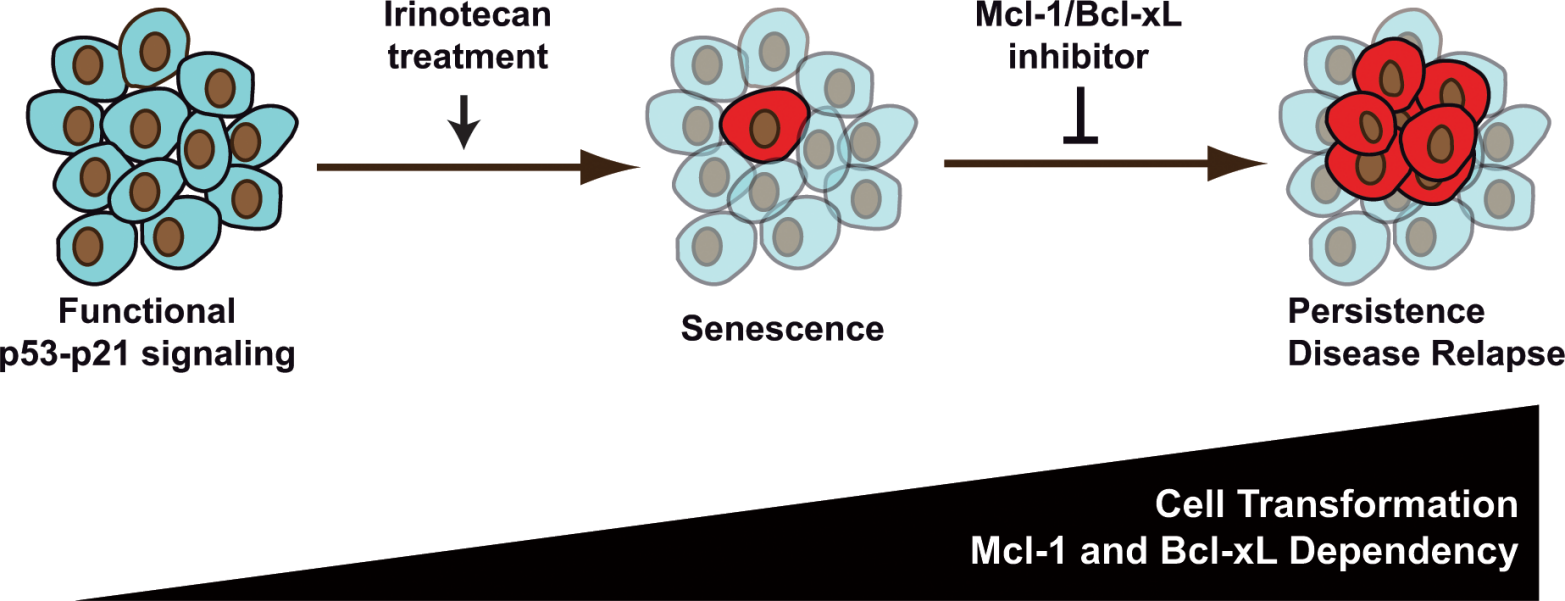
sn38 survival is associated with increased cell transformation and dependency on Mcl-1: an opportunity for new treatments in colorectal cancer In response to sn38, a small fraction of cells escapes the senescence suppressive arrest and emerges as an heterogeneous and more transformed population. We speculate that these cells are responsible of treatment failure and disease relapse. Since surviving cells depend on Mcl-1 signaling (and to a lesser extent on Bcl-xL), we propose that inhibitors of the Bcl2 family should be used to improve irinotecan efficiency in the treatment of colorectal cancers.

These adaptations illustrate the importance of tumor heterogeneity in treatment failure [44]. Although targeted therapies can reduce the activity of a deregulated oncogene, much less is known on how we could prevent the emergence of heterogeneous persistent cells in response to treatment. Future experiments should identify synthetically lethal drugs that could target specific subpopulations and allow a complete eradication of cancer cells when used in combination with irinotecan. The survival of resistant populations has been correlated with rescue pathways, such as the Met and IGFR receptors or secreted proteins of the Wnt and Hedgehog family [15, 45]. Since anti-EGFR targeted therapies such as cetuximab and panitunumab improve tumor response when used in combination with irinotecan in colorectal cancer [46, 47], it will be interesting to determine the effect of the EGF pathway and of these antibodies on PLCs emergence. In light of our results, we also propose the hypothesis that inhibitors of Mcl-1 and Bcl-xL should be tested in combination with irinotecan to prevent cell emergence (Figure 9). Phase I and phase II trials have already enrolled patients suffering from lymphoid malignancies or small-cell lung cancers to test the efficiency of BH3 mimetics such as ABT-263 or ABT-199 [32]. Inhibitors of Mcl-1 have been recently described but direct and non toxic molecules have yet to be tested in clinic [48]. We therefore propose the hypothesis that these inhibitors should be used in combination therapy to prevent emergence and reduce the occurrence of irinotecan-refractory colorectal carcinomas.

## MATERIALS AND METHODS

### Cell lines and treatment emergence

Colorectal cell lines (American Type Culture Collection) were maintained in antibiotic-free RPMI 1640 medium (Lonza), supplemented with 10% fetal bovine serum and maintained at 37 °C in 5% carbon dioxide. Cells were routinely tested for the absence of mycoplasma contamination. Unless indicated, note that cell treatments were performed in 3% FBS. For persistent cell generation, cells were treated for the indicated times with sn38 (5ng/ml; Tocris Bioscience 2684) in 3% FBS, washed with PBS and then restimulated with fresh 10% FBS. Proliferation generally resumed after 3-5 days. Other treatments were used : Salinomycin (1 or 5μM; Sigma Aldrich S6201) and ABT737 (1μM; Selleckchem S1002).

### Cell transfection, siRNA

RNA interference experiments were performed by transfection of 50 nM of prevalidated siRNA against Mcl-1 (Ambion AM51331 (ID:120642) or Cell signaling 6315), or control siRNA (MWG 5′-GCACUAACUACCGUGAUUATT-3′ and 5′-GAAAGAAGCACUCGUAUAATT-3′) using DharmaFect-4 (Dharmacon), according to the manufacturer's instructions.

### Clonogenic Assay

Assays were performed in control or treated cells as well as in PLCs to evaluate proliferation and survival. 500 to 1500 cells were seeded into 6-well cell culture plates with 10% FBS and incubated at 37°C in a 5% CO2 atmosphere for 7 to 10 days, then washed twice with PBS, and stained with 0.4% crystal violet. The colonies were then washed twice with water, visualized with a Bio-Rad Chemi Doc XRS Imaging device and counted using Quantity One imaging software (Bio-Rad).

### Soft Agar / ECM gel Assay

Cell preparation is a very sensitive step. Cells were plated the first day in 3% FBS and treated or not with sn38 after 24hr. Four days later, adherent cells were removed with trypsin for 10 minutes and resuspended in 10% FBS culture medium. If grown and treated in 10% FBS during these 5 days, note that parental LS174T cells grew in soft agar, whereas this was not the case in 3% FBS. After centrifugation, cells pellet was suspended with 10% FBS culture medium and KOVA slides (Hycor) were used for cell count. For each samples, a minimum of three counts were carried out using three different slides chambers. The average of these three counts was used to calculate the volume of cell suspension to obtain a 1.10^6^ cells/mL solution, in 100μL of 10% FBS culture medium. 5μL or 10μL of this, containing 5 000 or 10 000 cells, was transferred to 1,5mL tube. These cells were suspended in 100 μl of preheated agarose and then seeded on the lids of agar-coated 96-well plates. The base agar layer contained 50 μl of 1% agarose (Invitrogen) in 10% FBS culture medium with Penicillin-Streptomycin (Lonza). The 100 μl top agarose solution, containing the cells, was composed of 0,35% low melting point agarose (Promega) in 10% FBS culture medium with antibiotics. From one to three weeks after incubation at 37°C with 5% CO2, cell clones were counted and images were made. Every sample was tested in three technical replicates. Representative pictures were shown and the average of these three replicates was used for the clones count. For growth in ECM gel, the top agarose solution was substituted by half diluted ECM gel (Sigma) in 10% FBS culture medium with antibiotic.

### Tumor Xenografts

We conducted animal study in accordance with the guidelines approved by the Institutional Ethical Committee for Experimental Animal. Male Balb/c-nu mice were inoculated with one million cells subcutaneously in the right flank at the age of 8 to 9 weeks. Electronic caliper was used to measure the length (L), width (W) and depth (D) of tumor 2 to 3 times per week. Tumor volume was estimated by applying the following equation: 0,52 x L x W x D. Experiments were conducted using six mice per group.

### β-Galactosidase (β-Gal) staining

Cells were fixed for 15 min (room temperature) in 1% formaldehyde, washed with PBS and incubated at 37°C (no CO2) with fresh staining solution: 0.3 mg/mL of 5-bromo4-chloro-3-indolyl β-D-galactoside (X-Gal, Fermentas), 40 mM citric acid (Sigma), 40 mM sodium phosphate (Sigma) (stock solution (400 mM citric acid, 400 mM sodium phosphate) must be at pH6), 5 mM potassium ferrocyanide (Sigma), 5 mM potassium ferricyanide (Sigma), 150 mM NaCl (Sigma), 150 mM MgCl2 (Sigma). SA-β-GAL-positive cells were quantified after 16-20 hrs as compared to unstained cells.

### Flow Cytometry

*DAPI DNA staining:* Trypsinized cells were washed with PBS 2% BSA and 250 000 cells were fixed with 4% paraformaldehyde for 10 min at 37°C, then washed and incubated with cold 90% methanol at 4°C for 30 min. After washing, cells were then incubated with DAPI (5μg/ml) in PBS 2% BSA 0.2% Triton for one hour at room temperature. Cells were then analyzed by flow cytometry. Where indicated, vindelov staining was also used to analyze DNA content. *Ki67 staining :* Cells were trypsinized, washed with PBS and incubated with cold 70% methanol at 4°C for one hour. Cells were then washed twice with PBS Tween 0,1% and PBS Tween 0,1% BSA 1%. Then, cells were incubated 30 min at room temperature with mouse anti-human Ki-67(Dako M7240) or with mouse IgG1 (Beckman Coulter IM0571) as a control isotype. After washing, cells were incubated 30 min at room temperature with FITC goat anti-mouse IgG as secondary antibody. After washing, DAPI DNA staining was performed as described above. *BrdU staining* (APC BrdU Flow Kit; BD Pharmingen 552598) : BrdU was added to cell culture (10 μM) 4 hours before the end of the experiment. The following steps were performed according to the manufacturer's instructions. *pH2AX staining :* First steps were performed as described in «*DAPI DNA staining» procedure*. Before DNA staining, 250000 cells were incubated one hour at room temperature with 31 ng A488 mouse anti-pSer139 H2AX (BD Biosciences 560445) or with 31 ng A488 mouse IgG1k (BD Biosciences 557721) as control isotype control. *Extracellular staining :* Cells were harvested by chelating with PBS 2mM EDTA pH 8 and washed with PBS 2% BSA. 250 000 cells were incubated 15 min at room temperature with 12 ng APC mouse anti-CD44 (BD Biosciences 559942) or with 12 ng APC mouse IgG2bk (BD Biosciences 555745) as control isotype; or with 31 ng A647 rat anti-LGR5 (BD Biosciences 562903) or with 31 ng A647 rat IgG2bk (BD Biosciences 557691) as control isotype; or with 0.25μL PE mouse anti-CD133 (Miltenyi Biotec 130-098-826) or with 0,25μL PE mouse IgG1 (Miltenyi Biotec 130-098-845) as control isotype. After two washes, cells were analysed by FACS. *ALDH staining:* A kit was used for ALDH activity detection (Aldefluor Stemcell technologies 01700).

### FACS cell sorting

Cells were trypsinized, washed with PBS 1% FBS, filtered through a 40 μm sterile cell strainer and then processed using a FACSAria Cell Sorter (BD Biosciences).

### Western Blotting

The following antibodies were used: cleaved Caspase 3 (Asp175) (1/1000; Cell signaling 9661), Bim (1/1000; Millipore AB17003), Bax (1/1000; Cell signaling 2774), monoclonal anti-Bcl-2 (1:1000; Santa Cruz sc 7382 ), rabbit polyclonal anti-Mcl-1 (1:1000; Santa Cruz sc 819), rabbit monoclonal anti-p21 (1:1000; Cell Signaling 2947), mouse monoclonal anti-HSC70 (1:1000, Santa Cruz sc 7298).

### Quantitative PCR

Analyses were performed following DNase I treatment (Fermentas), using the comparative *C*T method (2^(ΔCt)), with normalization of the target gene expression to the endogenous housekeeping gene RPLPO or G3PDH. PCR primers sequences were as follows: RPLPO (5′-AACCCAGCTCTGGAGAAACT-3′ and 5′-CCCCTGGAGATTTTAGTGGT-3′), G3PDH (5′-GAAGGTGAAGGTCGGAGTC-3′ and 5-GAAGATGGTGATGGGATTTC-3′), Mcl-1 (5′-CGTAAGGACAAAACGGGACTGGC-3′ and 5′-ACCTGCAAAAGCCAGCAGCACA-3′), Bcl-xL (5′-GTAAACTGGGGTCGCATTGT-3′ and 5′-TGCTGCATTGTTCCCATAGA-3′), p21 (5′-GCTCCTTCCCATCGCTGTCA-3′ and 5′-TCACCCTGCCCAACCTTAGA-3′), AURKA (5′-GCCCTCTGGGTAAAGGAAAG-3′ and 5′-TCCAAGTGGTGCATATTCCA-3′) and PLK1 (5′-AAGAGATCCCGGAGGTCCTA-3′ and 5′-GCTGCGGTGAATGGATATTT-3′).

### Statistical Analysis

All values were expressed as mean +/− standard deviation (SD). Differences were analysed using non parametric tests (Mann-Whitney and Wilcoxon tests). *p < 0.05, **p < 0.01, and ***p < 0.001.

## Abbreviations

PLCs: Persistent LS174T Cells
